# Inconsistent self-reported mammography history: Findings from the National Population Health Survey longitudinal cohort

**DOI:** 10.1186/1472-6963-4-32

**Published:** 2004-11-12

**Authors:** Christina M Bancej, Colleen J Maxwell, Judy Snider

**Affiliations:** 1 Centre for Chronic Disease Prevention & Control, Population and Public Health Branch, Health Canada, Ottawa ON, Canada; 2 Departments of Community Health Sciences and Medicine & Fellow of the Institute of Health Economics, University of Calgary, Calgary AB, Canada; 3 Tobacco Control Programme, Healthy Environments and Consumer Safety Branch, Health Canada, Ottawa ON, Canada

## Abstract

**Background:**

Self-reported information has commonly been used to monitor mammography utilization across populations and time periods. However, longitudinal investigations regarding the prevalence and determinants of inconsistent responses over time and the impact of such responses on population screening estimates are lacking.

**Methods:**

Based on longitudinal panel data for a representative cohort of Canadian women aged 40+ years (n = 3,537) assessed in the 1994–95 (baseline) and 1996–97 (follow-up) National Population Health Survey (NPHS), we examined the prevalence of inconsistent self-reports of mammography utilization. Logistic regression models were used to estimate the associations between women's baseline sociodemographic and health characteristics and 2 types of inconsistent responses: (i) baseline reports of ever use which were subsequently contradicted by follow-up reports of never use; and (ii) baseline reports of never use which were contradicted by follow-up reports of use prior to 1994–95.

**Results:**

Among women who reported having a mammogram at baseline, 5.9% (95% confidence interval (CI): 4.6–7.3%) reported at follow-up that they had never had one. Multivariate logistic regression analyses showed that women with such inconsistent responses were more often outside target age groups, from low income households and less likely to report hormone replacement therapy and Pap smear use. Among women reporting never use at baseline and ever use at follow-up, 17.4% (95%CI: 11.7–23.1%) reported their most recent mammogram as occurring prior to 1994–95 (baseline) and such responses were more common among women aged 70+ years and those in poorer health.

**Conclusions:**

Women with inconsistent responses of type (i), i.e., ever users at baseline but never users at follow-up, appeared to exhibit characteristics typical of never users of mammography screening. Although limited by sample size, our preliminary analyses suggest that type (ii) responses are more likely to be the result of recall bias due to competing morbidity and age. Inconsistent responses, if removed from the analyses, may be a greater source of loss to follow-up than deaths/institutionalization or item non-response.

## Background

In the absence of organized screening, self-report is often the only means to monitor mammography utilization and to investigate trends in uptake at the population level [1]. In Canada, mammographic screening occurs both within organized programs and opportunistically through routine medical practice [2]. The validity of mammography self-report has previously been studied primarily using convenience samples. Despite differences in methodology, design and population characteristics, studies from a variety of settings have found that self-reports of mammography use are valid provided women are not required to precisely recall the timing of a previous mammogram [3-14]. Women generally tend to underestimate the time elapsed since their most recent mammogram by an average of three months or more, though overestimation can also occur [4-6,8-13,15-17]. Greater discrepancies in recall may occur when the mammogram took place longer ago [15,16], though contrary evidence also exists [11]. With some exceptions [4,6,8,9,13,16,17], studies have not been designed to assess false negative reporting due to the challenge of verifying historical data from multiple service providers. Those that have attempted to validate non-use have indicated that women are unlikely to deny having had a mammogram when indeed they have had one [4,6,8,9,13,17], though false-negative self-reports are not always negligible [16]. However, the opposite is often true; women tend to report having had mammograms which are not verified against medical records [3,4,6,7,9-11,13-16,18-21], a particular problem in groups with low screening prevalence [20]. Valid reporting of mammography use has been found to be unrelated to various health behaviors and perceptions, socioeconomic, demographic, and questionnaire administration factors in some studies [4,10,12,13]. However, others provide some evidence that age, ethnicity, education, employment status, family history of breast cancer, recency of the mammogram, and the regularity with which women receive mammograms do affect self-report accuracy [7,10-13,17,22].

Few evaluations of the reliability of mammography self-report are reported in the literature. Excellent test-retest reliability for having ever had a mammogram was reported in interviews conducted 1 week, 6–30 days, or 6–8 months after an initial interview, while reliability within the past year varied from excellent to good [14,23,24]. In a socioeconomically advantaged group of women aged 50–75 followed annually for 3 years, 98 percent provided logically consistent responses to a question on ever/never use of mammography [25]. Self-reports of ever use have been shown to be more reliable among Caucasian women and those with higher income and education [24]. However, in this study, date of last mammogram was not as reliably reported 6–8 months after initial testing [24].

Based on data from the longitudinal panel of the National Population Health Survey (NPHS), the present study examines the prevalence and determinants of inconsistent self-reports of mammography utilization among Canadian women aged 40 years and older and quantifies the extent that inconsistent self-reports of mammography use contribute to biased estimates of mammography utilization and uptake. To our knowledge, this is one of the first studies of mammography utilization to provide specific longitudinal data on the determinants of inconsistent responses over time and the impact of such responses on population screening estimates.

## Methods

The National Population Health Survey (NPHS) is a survey of the Canadian household population. Initiated in 1994–95 and repeated biennially, it is a split panel survey, combining repeated cross-sectional components with the longitudinal follow-up of a panel of respondents. A representative sample of Canadian household residents aged 12 and older from all ten provinces was sampled using a multistage probability design with stratification and clustering at various stages. The overall response rate for the baseline 1994–95 survey was 89 percent with a 96 percent response rate for the selected panel respondent [26]. On follow-up to the baseline survey two years later, 94 percent of the panel members responded [26]. Further details of the sampling procedures, design, data collection and response rates are published elsewhere [26,27].

This study evaluated data from longitudinal panel respondents of the 1994–95 (baseline) and 1996–97 (follow-up) waves of the NPHS to examine inconsistencies in reported mammography utilization among women aged 40+ years at first contact. Questions about mammography use were administered to female respondents through a personal interview conducted in 1994–95 and repeated by telephone approximately two years later. In both survey years, women were asked the identical question: "Have you ever had a mammogram, that is, a breast x-ray?". Those with positive responses were further probed for the time and reason of their most recent mammogram. All women provided their own health-related information; no proxy responses were allowed.

Analyses were restricted to women aged 40 and older (at baseline) who participated in the first two waves of the NPHS and consented to share their information with federal and provincial governments. Two types of inconsistent responses were assessed: (i) baseline reports of ever use which were contradicted by follow-up reports of never use; and (ii) baseline reports of never use which were contradicted by follow-up reports of use prior to 1994–95. Multivariate logistic regression techniques were used to evaluate the associations between women's baseline sociodemographic and health characteristics and type (i) inconsistent responses. Variables significant at p ≤ .05 in age-adjusted analyses were eligible for entry in the multivariate logistic models. Sample size constraints permitted only simple bivariate, rather than multivariate exploration of factors associated with reports reflecting inconsistent timing of most recent use at follow-up (type (ii) response). Estimates were weighted to reflect baseline population characteristics. To account for stratification and clustering in the NPHS sampling design, 95% confidence intervals for parameter estimates were calculated using exact standard errors generated through bootstrap re-sampling methods [28]. All statistical analyses were conducted using SAS.

## Results

### (i) Inconsistent ever/never utilization

**Figure 1:**
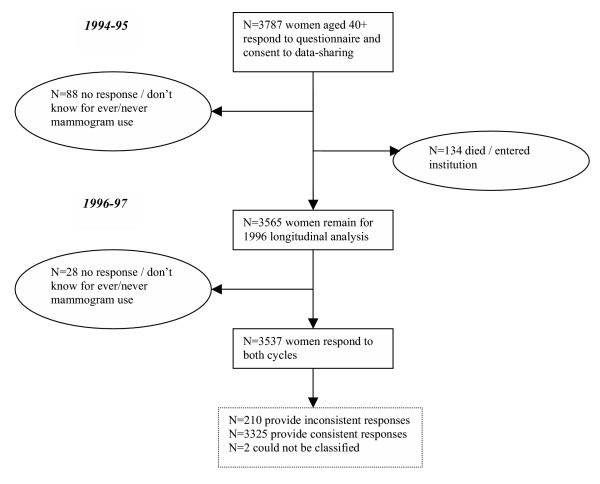
Response characteristics of the NPHS longitudinal panel of women aged 40+ years in 1994–95.

Of the 3,535 women aged 40+ years who responded to the ever/never mammography question in both survey waves (Figure 1: 2 women with missing data regarding timing of mammogram were excluded), four percent (95% CI: 3.1–4.9) reported having had a mammogram at baseline and subsequently, on follow-up reported never having had a mammogram (Table 1). Among women who reported having had a mammogram at baseline, 5.9% (95%CI: 4.6–7.3) reported never use at follow-up (estimate not shown). The majority of women with inconsistent responses (64.4%, 95% CI: 54.4–74.4) reported receiving a recent (i.e., <2 years ago) mammogram at baseline and most (85.6%, 95% CI: 78.2–93.1) reported that the mammogram was done as part of a regular check up (Table 1). It should be noted that the percentage estimates in Table 1 have been *weighted *according to 1994/95 population characteristics whereas the frequency data represent actual numbers of women surveyed.

Table 2 presents the estimated adjusted odds ratios (95% CIs) of inconsistent ever/never responses associated with women's baseline sociodemographic and health characteristics. Among women reporting ever use at baseline (1994–95), those reporting never use in 1996–97 were significantly more likely to be outside the target age group for screening (50–69), to have lower income, to have not used hormone replacement therapy in the past month and to have never had a Pap test, after adjusting for relevant covariates. Women with lower education levels were also more likely to report such inconsistent responses between baseline and follow-up although education failed to remain a significant predictor in the multivariate model. Other variables considered but not found to be significantly associated with this outcome were rural/urban residence, place of birth, languages spoken, marital status and other social support indices and having a regular physician.

### (ii) Inconsistent timing

Follow-up interviews were completed, on average, 1.98 years from the baseline survey (range 1.19–3.01 years). Of the 293 women who reported never use at baseline and ever use at follow-up, 17.4 percent (95%CI: 11.7–23.1) reported a time for their most recent mammogram at follow-up that was inconsistent with never use at baseline. Despite baseline reports that they had never had a mammogram, approximately half of these women reported having had a mammogram at least 5 years ago. Although limited by small numbers, determinants of such inconsistent responses were assessed with simple bivariate analyses. Inconsistencies in timing occurred more often in older women. Compared to women aged 50–69, those 70 and older were more likely to report (at follow-up) that their most recent mammogram had occurred prior to 1994–95, despite a report of never use at baseline (OR = 6.96, 95%CI: 2.42–20.0). Women reporting fair or poor self-rated health were also more likely to report a time for their most recent mammogram at follow-up that was inconsistent with never use at baseline (OR = 2.44, 95% CI: 0.99–6.05).

#### Impact of inconsistent reporting on uptake estimates

Depending on how inconsistent responses are handled, different measures of use and uptake of mammography may be obtained. The lack of a gold standard such as a medical chart for validation makes the choice of a corrective measure unclear. If inconsistent ever/never responses are included in the analysis unchanged, 67.3 percent (95% CI: 65.1–69.5%) of women would be classified as ever having had a mammogram in 1994–95 while 71.7 percent (95% CI: 69.6–73.7%) would be classified as ever users in 1996–97. Conversely, if it is assumed that inconsistent ever/never responses represent false-positive responses at baseline (an assumption supported by our study findings), the 1994–95 prevalence estimate becomes 63.3 percent (95% CI: 61.0–65.6%), demonstrating an absolute increase in mammography use of 8.4 percent (95% CI: 7.1–9.6%) by this cohort of women by 1996–97.

## Discussion

Although a limited number of studies have assessed the reliability of mammography self-report [23-25], detailed evaluations have not been conducted for population-based longitudinal surveys. In this study, reliability could not be assessed, per se, as women's status of never having had a mammogram could normally be expected to change over a two year span. However, by examining inconsistencies in responses expected to remain constant and in responses regarding logical timing of mammography use, it is possible to examine potential concerns regarding response reliability and recall bias, respectively. In longitudinal studies, inconsistent data removed during data cleaning can yield significant losses, and may lead to bias, depending on the amount of attrition at each time point and the magnitude of the differences between those retained in the panel and those lost by such attrition [29]. Longitudinal studies of health must be acutely aware of causes of attrition because losses accumulate over survey waves [30].

Although direct comparison with our sample was not possible due to the expectation that behavior might have changed in a 2-year time span, earlier findings from longitudinal studies of fairly affluent [25] and low-income [24] populations and a population-based study [23], indicated that women reliably report having ever had a mammogram with estimated reliability measured by Cohen's kappa ranging from 0.82–0.87 [23,24]. Our finding that 4 percent (95% CI: 3.1–4.9 percent) of the women participating in the second wave of this longitudinal study inconsistently reported ever having had a previous mammogram was surprisingly high. Previous studies have found that initial use refuted on subsequent interviews occurred in 2–2.9 percent of respondents [24,25].

Our analyses of factors associated with inconsistent ever/never responses indicate that women reporting ever having had a mammogram at baseline but never use at follow-up exhibited many of the sociodemographic and health behaviour characteristics (e.g., lower income, outside age groups targeted for screening, non-users of Pap screening and hormone replacement therapy) commonly observed among non-participants in mammography screening in previous studies [31-38]. Such findings provide support for the assumption that the 1994–95 response of ever use is more likely erroneous. Additional factors (e.g. being born in an Asian country) previously associated with non-use of mammography [31,33] also showed a positive association with providing an inconsistent response; however, small numbers resulted in high variability once clustering and stratification were taken into account and precluded further analysis of this variable.

Validation studies also provide support for our assumption that inconsistent ever/never responses (as assessed in the present longitudinal panel) are most likely to be explained by false-positive responses at baseline. Women are more likely to falsely claim having had a mammogram, than not having one [4,6,8,9,13,17,39,40]. The majority of women in our study with inconsistent ever/never responses also indicated (at baseline) that their most recent mammogram had occurred within the last two years, a finding consistent with past research [10]. Imputation, suggested as a remedy for item non-response [30], may equally be used to deal with inconsistencies with evidence, in this situation, favoring treatment of women's earlier responses as false-positive.

Among women who reported never use at baseline and ever use at follow-up, approximately 17.4 percent reported a time for their most recent mammogram (at follow-up) that was inconsistent with never use at baseline. If respondents truly initiated mammography use subsequent to the baseline interview, they overestimated the time elapsed since their mammogram. Such a finding is relatively inconsistent with previous studies that have generally found that women tend to underestimate the time since their last mammogram [4-6,8-13,15,17]. Although McGovern et al. and Caplan et al. found reverse-telescoping in approximately 9 percent of their samples [13,16], only 8 percent of women in this group miscalculated by more than 1 year [16]. In the present study, women reporting inconsistent timing were older and in poorer health, suggesting that competing health events may have interfered with accurate recall.

Unfortunately, no gold standard was available to assess the validity of the responses women generated. Thus, the proportion of consistent responses that may actually represent invalid responses is unclear. Nor was it possible to distinguish errors in recall of timing from false reports of mammography utilization. Further, the reasons for providing inconsistent responses can only be inferred. The possibility of data entry errors is remote. Although data entry checks for consistency between survey cycles were not included among the comprehensive quality control strategies implemented by Statistics Canada, computer assisted interviewing was used by highly trained interviewers. Also, data entry errors would be expected to occur randomly and not disproportionately among women outside of the target age range or with relatively lower socioeconomic status, as observed in the present study. However, several plausible explanations for the inconsistencies exist, including survey methodology changes and deliberate or inadvertent provision of inaccurate responses by the respondent.

We cannot exclude the possibility that interview changes (from a baseline personal interview to a telephone follow-up) prompted women who reported ever use in 1994/95 to alter their response in 1996–97. The NPHS used an initial personal interview to foster a good long-term relationship with the panel representative, but the cost and logistics of traveling to different regions was prohibitive. Therefore, unless the respondent objected or had no phone, future interviews (including the 1996–97 cycle) were conducted by telephone [26]. The need to maintain study procedures over time in longitudinal studies has been stressed [41], but the impact of altering the interview method on the NPHS results has not been investigated [26]. Sensitive questions may be answered more truthfully by phone. Editing of survey responses by the respondent may occur. Social desirability and a tendency to give positive responses are possible sources of over-reporting [3,11]. Such biases remain largely unexplored with respect to mammography use.

Cognitive research has implicated comprehension as a barrier to providing valid, reliable survey responses. Several researchers employ the lead-in question 'Have you ever heard of a mammogram, that is a breast x-ray?' to identify comprehension difficulties [3,16,21,42] but this was not done in the NPHS where women were directly asked if they had ever had a mammogram. One study using focus group testing and in-depth interviews showed that despite some confusion between mammography and breast exams, women generally understood what mammography was [11], a finding further supported by one population-based survey [21]. However other investigations suggest this is not uniformly so [16,42,43]. One possibility cited is that confusion with other tests such as chest x-rays may lead to over-reporting of mammography [3].

There are several possible explanations for the higher rate of inconsistent responses observed in the present study. The focus of the NPHS questionnaire was not limited to preventive practices nor was it designed for reliability testing. The length and comprehensiveness of the NPHS may have contributed to greater respondent fatigue. Also, the longer time interval between surveys, relative to that observed in other studies, may have contributed to instability in responses. A more favourable level of concordance among responses may be obtained by studies that apply eligibility criteria that ensure more accurate responses (e.g. by having the respondent recall where she had her mammogram to validate her ever/never use) [6,10,15]. Finally, the overall response rate of the NPHS was higher than in comparable studies, so it potentially included a more difficult-to-reach population, less able to provide accurate responses.

The inconsistent data reported here, if removed from longitudinal analyses, could yield losses to follow-up equivalent to or greater than other sources of attrition (e.g., deaths, institutionalization, non-response) over the planned 20 year course of the NPHS. The 1998–99 NPHS included probing questions to reduce inconsistencies and it should alleviate many of the problems evident here [44]. However, probes were not designed to address the larger problem of reverse-telescoping observed among some respondents in the NPHS longitudinal cohort. Incorporation of women's previous responses in subsequent interviews to avoid telescoping and stimulate recall can be used to minimize such inconsistencies [30]. A recent critical review of the accuracy of self-reported health behaviors, including mammography, provides further suggestions for enhancing the accuracy of such data [45].

## Conclusions

In summary, inconsistent responses represent a challenge to longitudinal, population-based evaluations of breast screening practices. Losses from inconsistent data regarding mammography participation are not negligible and may contribute to inaccurate estimates of mammography uptake. Women reporting inconsistent ever/never use in the present study displayed characteristics typical of never users, favoring treatment of women's baseline responses as false-positive. Inconsistent responses regarding the timing of recent mammography practices, however, may be primarily related to the impact of age and competing morbidity on recall.

## Competing interests

The authors declare that they have no competing interests.

## Authors' contributions

CB and CM contributed to the initial and revised analyses of the NPHS panel data, the interpretation of the results and the initial drafting/editing of the manuscript. CB and JS were responsible for the data linkage with Statistics Canada (for the NPHS Share File) and for the revised bootstrap analyses. JS also contributed to the interpretation of the results and editing of the manuscript.

## Pre-publication history

The pre-publication history for this paper can be accessed here:



## Figures and Tables

**Table 1:** Mammography utilization characteristics among Canadian women aged 40+ years, 1994–95 and 1996–97 NPHS longitudinal cohort (n = 3535)*

**Characteristic**	**Frequency**	**Percent (95% CI)**^†^
**Consistent Responders**	**3325**	**94.5 (93.5–95.6)**
Ever had mammogram (1994–95) and (1996–97)	2097	63.3 (61.0–65.6)
Never had mammogram (1994–95) and (1996–97)	987	24.3 (22.4–26.3)
Never had mammogram (1994–95), Ever had (1996–97)^‡^	241	6.9 (5.7–8.1)
**Inconsistent Responders**	**210**	**5.5 (4.4–6.5)**
Never had mammogram (1994–95), Ever had (1996–97)^§^	52	1.5 (1.0–2.0)
Ever had mammogram (1994–95), Never had (1996–97)	158	4.0 (3.1–4.9)
*Time since last mammogram reported in 1994–95*		
*< 6 months*	*32*	*20.3 (9.9–30.7)*
*≥ 6 months and < 2 years*	*55*	*44.1 (33.1–55.0)*
*≥ 2 years*	*71*	*35.6 (25.6–45.6)*
*Reason for mammogram: check up / no particular problem*	*132*	*85.6 (78.2–93.1)*

* n = 2 respondents not classified as consistent or inconsistent as they reported initiation of mammography use in 1996–97 but did not report time of most recent mammogram

† weighted according to 1994–95 population characteristics

‡ reported time of mammogram consistent with interval between surveys

§reported time of mammogram inconsistent with interval between surveys

**Table 2:** Estimated Odds Ratios (95% CIs) of inconsistent responses for ever having had amammogram* according to baseline sociodemographic and health characteristics amongCanadian women (aged 40+) assessed in the 1994–95 and 1996–97 NPHS (n = 2255).

**Characteristic**	**Age-Adjusted OR (95%CI)**	**Adjusted OR^† ^(95%CI)**
Age Group		
40–49	2.40 (1.41–4.09)	2.11 (1.15–3.88)
50–69	1.00	1.00
70+	3.62 (2.05–6.38)	3.18 (1.83–5.54)
Household Income		
not stated	2.02 (0.48–8.49)	1.92 (0.51–7.21)
low ($0–19,999)	1.95 (1.16–3.28)	1.84 (1.04–3.24)
moderate ($20,000–59,999)	1.00	1.00
high ($60,000+)	0.32 (0.10–0.96)	0.34 (0.11–1.05)
Educational Attainment		
elementary / some secondary	1.00	1.00
secondary graduate / some post secondary	0.60 (0.34–1.05)	0.80 (0.45–1.42)
post-secondary degree	0.39 (0.21–0.72)	0.59 (0.31–1.13)
Hormone drug use (past month)		
no	1.00	1.00
yes	0.41 (0.24–0.71)	0.44 (0.25–0.78)
Ever had Pap test		
yes	1.00	1.00
no	3.19 (1.52–6.68)	2.82 (1.39–5.72)

* refers to women reporting yes to question on ever having had a mammogram in 1994–95 survey and no in 1996–97 survey; comparison group represents women reporting yes in both surveys

† obtained from multivariate logistic regression model, adjusted for all variables listed in table; other variables examined but not found to be significant predictors of inconsistent ever/never responses were: rural residence, place of birth, language, marital status and having a regular physician

## References

[B1] World Health Organization (1995). National cancer control programmes: policies and managerial guidelines.

[B2] Gaudette LA, Altmayer CA, Nobrega KM, Lee J (1996). Trends in mammography utilization, 1981 to 1994. Health Rep.

[B3] Champion VL, Menon U, McQuillen DH, Scott C (1998). Validity of self-reported mammography in low-income African-American women. Am J Prev Med.

[B4] Degnan D, Harris R, Ranney J, Quade D, Earp JA, Gonzalez J (1992). Measuring the use of mammography: two methods compared. Am J Public Health.

[B5] Etzi S, Lane DS, Grimson R (1994). The use of mammography vans by low-income women: the accuracy of self-reports. Am J Public Health.

[B6] Gordon NP, Hiatt RA, Lampert DI (1993). Concordance of self-reported data and medical record audit for six cancer screening procedures. J Natl Cancer Inst.

[B7] Hiatt RA, Perez-Stable EJ, Quesenberry C, Sabogal F, Otero-Sabogal R, McPhee SJ (1995). Agreement between self-reported early cancer detection practices and medical audits among Hispanic and non-Hispanic white health plan members in northern California. Prev Med.

[B8] King ES, Rimer BK, Trock B, Balshem A, Engstrom P (1990). How valid are mammography self-reports?. Am J Public Health.

[B9] Paskett ED, Tatum CM, Mack DW, Hoen H, Case LD, Velez R (1996). Validation of self-reported breast and cervical cancer screening tests among low-income minority women. Cancer Epidemiol Biomarkers Prev.

[B10] Suarez L, Goldman DA, Weiss NS (1995). Validity of Pap smear and mammogram self-reports in a low-income Hispanic population. Am J Prev Med.

[B11] Warnecke RB, Sudman S, Johnson TP, O'Rourke D, Davis AM, Jobe JB (1997). Cognitive aspects of recalling and reporting health-related events: Papanicolaou smears, clinical breast examinations, and mammograms. Am J Epidemiol.

[B12] Zapka JG, Bigelow C, Hurley T, Ford LD, Egelhofer J, Cloud WM, Sachsse E (1996). Mammography use among sociodemographically diverse women: the accuracy of self-report. Am J Public Health.

[B13] Caplan LS, Mandelson MT, Anderson LA (2003). Validity of self-reported mammography: examining recall and covariates among older women in a Health Maintenance Organization. Am J Epidemiol.

[B14] Barratt A, Cockburn J, Smith D, Redman S (2000). Reliability and validity of women's recall of mammographic screening. Aust N Z J Public Health.

[B15] Fulton-Kehoe D, Burg MA, Lane DS (1992). Are self-reported dates of mammograms accurate?. Public Health Rev.

[B16] McGovern PG, Lurie N, Margolis KL, Slater JS (1998). Accuracy of self-report of mammography and Pap smear in a low-income urban population. Am J Prev Med.

[B17] Caplan LS, McQueen DV, Qualters JR, Leff M, Garrett C, Calonge N (2003). Validity of women's self-reports of cancer screening test utilization in a managed care population. Cancer Epidemiol Biomarkers Prev.

[B18] May DS, Trontell AE (1998). Mammography use by elderly women: a methodological comparison of two national data sources. Ann Epidemiol.

[B19] Montano DE, Phillips WR (1995). Cancer screening by primary care physicians: a comparison of rates obtained from physician self-report, patient survey, and chart audit. Am J Public Health.

[B20] Whitman S, Lacey L, Ansell D, Chen EH, Dell J, Phillips CW (1993). Do chart reviews and interviews provide the same information about breast and cervical cancer screening?. Int J Epidemiol.

[B21] Kottke TE, Trapp MA, Fores MM, Kelly AW, Jung SH, Novotny PJ, Panser LA (1995). Cancer screening behaviors and attitudes of women in southeastern Minnesota. JAMA.

[B22] Lawrence VA, De Moor C, Glenn ME (1999). Systematic differences in validity of self-reported mammography behavior: A problem for intergroup comparisons?. Prev Med.

[B23] Brownson RC, Jackson-Thompson J, Wilkerson JC, Kiani F (1994). Reliability of information on chronic disease risk factors collected in the Missouri Behavioral Risk Factor Surveillance System. Epidemiology.

[B24] Vacek PM, Mickey RM, Worden JK (1997). Reliability of self-reported breast screening information in a survey of lower income women. Prev Med.

[B25] Caplan LS, Lane DS, Grimson R (1995). The use of cohort vs repeated cross-sectional sample survey data in monitoring changing breast cancer screening practices. Prev Med.

[B26] Swain L, Catlin G, Beaudet MP (1999). The National Population Health Survey – its longitudinal nature. Health Rep.

[B27] Tambay JL, Catlin G (1995). Sample design of the national population health survey. Health Rep.

[B28] Rust KF, Rao JN (1996). Variance estimation for complex surveys using replication techniques. Stat Methods Med Res.

[B29] Curtin L, Feinleib M, Dwyer JH, Feinleib M, Lippert P (1992). Considerations in the design of longitudinal surveys of health. In Statistical Models for Longitudinal Studies of Health.

[B30] Duncan GJ, Kalton G (1987). Issues of design and analysis of surveys across time. International Statistical Review.

[B31] Maxwell CJ, Bancej CM, Snider J (2001). Predictors of mammography use among Canadian women aged 50–69: findings from the 1996/97 National Population Health Survey. CMAJ.

[B32] Maxwell CJ, Kozak JF, Desjardins-Denault SD, Parboosingh J (1997). Factors important in promoting mammography screening among Canadian women. Can J Public Health.

[B33] Hsia J, Kemper E, Kiefe C, Zapka J, Sofaer S, Pettinger M, Bowen D, Limacher M, Lillington L, Mason E (2000). The importance of health insurance as a determinant of cancer screening: evidence from the Women's Health Initiative. Prev Med.

[B34] Lagerlund M, Sparen P, Thurfjell E, Ekbom A, Lambe M (2000). Predictors of non-attendance in a population-based mammography screening programme; socio-demographic factors and aspects of health behaviour. Eur J Cancer Prev.

[B35] Anderson LM, May DS (1995). Has the use of cervical, breast, and colorectal cancer screening increased in the United States?. Am J Public Health.

[B36] Breen N, Kessler L (1994). Changes in the use of screening mammography: evidence from the 1987 and 1990 National Health Interview Surveys. Am J Public Health.

[B37] Calle EE, Flanders WD, Thun MJ, Martin LM (1993). Demographic predictors of mammography and Pap smear screening in US women. Am J Public Health.

[B38] Katz SJ, Hofer TP (1994). Socioeconomic disparities in preventive care persist despite universal coverage. Breast and cervical cancer screening in Ontario and the United States. JAMA.

[B39] Thompson BL, O'Connor P, Boyle R, Hindmarsh M, Salem N, Simmons KW, Wagner E, Oswald J, Smith SM (2001). Measuring clinical performance: comparison and validity of telephone survey and administrative data. Health Serv Res.

[B40] Martin LM, Leff M, Calonge N, Garrett C, Nelson DE (2000). Validation of self-reported chronic conditions and health services in a managed care population. Am J Prev Med.

[B41] Cook NR, Ware JH (1983). Design and analysis methods for longitudinal research. Annu Rev Public Health.

[B42] Mah Z, Bryant H (1992). Age as a factor in breast cancer knowledge, attitudes and screening behaviour. CMAJ.

[B43] Kindree T, Ashbury FD, Goel V, Levy I, Lipskie T, Futcher R (1997). Development of an instrument to measure cancer screening knowledge, attitudes and behaviours. Chronic Dis Can.

[B44] Swain L, Catlin G (1998). The National Population Health Survey: Its longitudinal nature. In Proceedings of the Joint IASS/IAOS Conference.

[B45] Newell SA, Girgis A, Sanson-Fisher RW, Savolainen NJ (1999). The accuracy of self-reported health behaviors and risk factors relating to cancer and cardiovascular disease in the general population: a critical review. Am J Prev Med.

